# Computed tomography volumetric analysis for predicting postoperative lung function for segmentectomy

**DOI:** 10.1093/icvts/ivac195

**Published:** 2022-07-25

**Authors:** Seon Yong Bae, Haeju Lee, Kwon Joong Na, Bubse Na, Samina Park, In Kyu Park, Chang Hyun Kang, Young Tae Kim

**Affiliations:** Department of Thoracic and Cardiovascular Surgery, Seoul National University Hospital, Seoul National University College of Medicine, Seoul, Korea; Department of Thoracic and Cardiovascular Surgery, Seoul National University Hospital, Seoul National University College of Medicine, Seoul, Korea; Department of Thoracic and Cardiovascular Surgery, Seoul National University Hospital, Seoul National University College of Medicine, Seoul, Korea; Cancer Research Institute, Seoul National University College of Medicine, Seoul, Korea; Department of Thoracic and Cardiovascular Surgery, Seoul National University Hospital, Seoul National University College of Medicine, Seoul, Korea; Department of Thoracic and Cardiovascular Surgery, Seoul National University Hospital, Seoul National University College of Medicine, Seoul, Korea; Department of Thoracic and Cardiovascular Surgery, Seoul National University Hospital, Seoul National University College of Medicine, Seoul, Korea; Department of Thoracic and Cardiovascular Surgery, Seoul National University Hospital, Seoul National University College of Medicine, Seoul, Korea; Department of Thoracic and Cardiovascular Surgery, Seoul National University Hospital, Seoul National University College of Medicine, Seoul, Korea; Cancer Research Institute, Seoul National University College of Medicine, Seoul, Korea

**Keywords:** Computed tomography, Lung cancer, Lung resection surgery, Predictive postoperative lung function

## Abstract

**OBJECTIVES:**

We compared the computed tomographic (CT) volumetric analysis and anatomical segment counting (ASC) for predicting postoperative forced expiratory volume in 1 s (FEV_1_) and diffusing capacity for carbon monoxide (D_LCO_) in patients who had segmentectomy for early-stage lung cancer.

**METHODS:**

A total of 175 patients who had segmentectomy for lung cancer and had postoperative pulmonary function test were included. CT volumetric analysis was performed by software, which could measure total lung and target segment volume from CT images. ASC and CT volumetric analysis were used to determine predicted postoperative (PPO) values and the concordance and difference of these values were assessed. The relationship between PPO values and actual postoperative values was also investigated.

**RESULTS:**

The PPO-FEV_1_ and PPO-D_LCO_ showed high concordance between 2 methods (concordance correlation coefficient = 0.96 for PPO-FEV_1_ and 0.95 for PPO-D_LCO_). There was no significant difference between PPO values as determined by 2 methods (*P* = 0.53 for PPO-FEV_1_, *P* = 0.25 for PPO-D_LCO_) and actual postoperative values [*P* = 0.77 (ASC versus actual) and *P* = 0.20 (CT versus actual) for FEV_1_; *P* = 0.41 (ASC versus actual) and *P* = 0.80 (CT versus actual) for D_LCO_]. We subdivided the patients according to poor pulmonary function test, the number of resected segments and the location of the resected lobe. All subgroup analyses revealed no significant difference between PPO values and actual postoperative values.

**CONCLUSIONS:**

Both CT volumetric analysis and ASC showed high predictability for actual postoperative FEV_1_ and D_LCO_ in segmentectomy.

**Figure:**
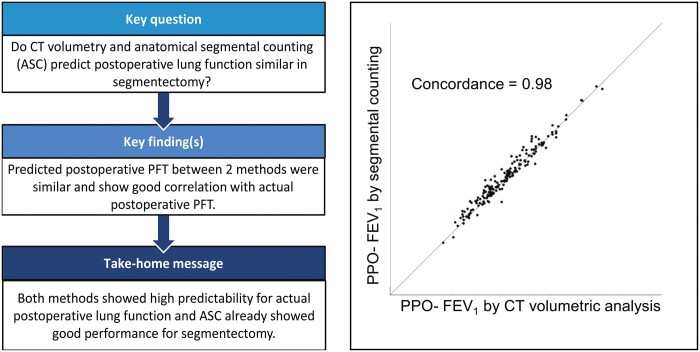

## INTRODUCTION

Lung cancer is the leading cause of cancer-related mortality worldwide, accounting for one out of every 5 deaths [1]. Surgical resection is mostly recommended for early-stage lung cancer and estimating predicted postoperative (PPO) pulmonary function test (PFT) is critical to predict a patient’s quality of life and prognosis, according to the American College of Chest Physicians (ACCP) guideline [2]. Both forced expiratory volume in 1 s (FEV_1_) and the diffusing capacity for carbon monoxide (D_LCO_) are key factors for physiologic evaluation before lung resection surgery. The conventional approach for estimating PPO-PFT is anatomical segment counting (ASC), which is based on counting the number of functional segments to be resected. Nevertheless, because the accuracy of this approach varies depending on the kind of surgery, numerous alternatives have been offered, including lung perfusion scintigraphy and computed tomography (CT) volumetric analysis [2–4].

Especially, CT volumetric analysis using high-quality CT and modelling software has been examined for predicting postoperative PFT [4]. In previous investigations, CT volumetric analysis was found to be more accurate than ASC in predicting PPO-FEV1 and PPO-DLCO after lobectomy, bilobectomy and pneumonectomy [4,5]. Because segmentectomy’s long-term oncologic prognosis in early-stage lung cancer was found to be comparable to that of lobectomy, segmentectomy has lately been advocated as an alternative surgical approach for treating early-stage lung cancer [6]. Despite the fact that segmentectomy is one of the most commonly used surgical procedures, there has been no research on a valid approach for calculating PPO-FEV1 and PPO-DLCO in segmentectomy.

The purpose of this study is to (i) compare PPO-FEV_1_ and PPO-D_LCO_ which is measured by ASC and CT volumetric analysis and (ii) compare these PPO-FEV_1_ and PPO-D_LCO_ with actual postoperative FEV_1_ and D_LCO_ in patients who underwent segmentectomy for early-stage lung cancer.

## MATERIALS AND METHODS

### Ethical statement

The study was approved by the Institutional Review Board of Seoul National University Hospital. The requirement for individual consent was waived (approval no., 2012-078-1181).

### Patients

We reviewed the medical records of 291 patients who underwent segmentectomy for clinically N0M0 lung cancer (American Joint Committee on Cancer 8th edition) between January 2010 and April 2017 at our institution. We excluded the patients (i) who did not perform postoperative PFT, (ii) who did not undergo a preoperative thin-section CT scan and (iii) whose preoperative CT was unable to reconstruct with the programme. As a result, a total of 175 patients were included for the analysis.

### Pulmonary function test

Patients underwent the PFT measurements using a spirometer (SPFS/D, MedGraphics, St. Paul, MN, USA) with the patients sitting in an upright position, based on the American Thoracic Society criteria [7]. PFT measurements included forced vital capacity (FVC), FEV_1_ and D_LCO_. The preoperative PFT was done within 1 month before the operation and the postoperative PFT was done about 6 months after surgery.

### Operation

In our centre, we mostly perform segmentectomy for patients with early-stage lung cancer or patients with limited pulmonary function or severe comorbidities. The indications for segmentectomy were decided by the individual physician. The segmental plane was identified by the use of ventilation after the segmental bronchus was transected. Subsequently, we marked the intersegmental plane and divided it with surgical staplers [8]. The parenchymal resection margin was confirmed based on pathologic results in the setting of a deflated lung. We subdivided segmentectomy into 2 groups, simple and complex, according to the surgical procedure and condition of the intersegmental plane. Segmentectomy that creates one, linear intersegmental plane, with a relatively easier procedure, could be considered as simple segmentectomy. Segmentectomy that creates several, intricate intersegmental planes, with a more complicated procedure, could be considered complex segmentectomy [9].

### Anatomical segment counting

PPO-PFT was calculated based on the ratio of functioning segments of lung using the following formula, proposed method by Nakahara *et al.* [10]. The lungs have the following 19 segments: right upper lobe (3 segments), right middle lobe (2 segments), right lower lobe (5 segments), left upper division segment (2 segments), lingual (2 segments) and left lower lobe (5 segments) [2,11].

PPO-PFT=preoperative PFT ×No. of remained segmentsNo. of segments 

.

### CT volumetric analysis

The three-dimensional image analysis system (SYNAPSE VINCENT, Fuji Film Co, Ltd, Tokyo, Japan) was used to obtain three-dimensional images from the tracheobronchial tree and calculated lung volume based on CT [4,12]. After manually designating resected segments of bronchus, the SYNAPSE calculated the volume of designated segment and total lung of individual patients. Postoperative CT was performed about 6 months after surgery. Finally, we calculated PPO-PFT by written formula.

PPO-PFT=preoperative PFT ×total lung volume-resected lung volumetotal lung volume.

### Statistical analysis

All statistical analyses were operated by using the R software package, version 3.4.3 (http://www.R-project.org). We calculated Lin’s concordance correlation coefficient (CCC) along with the 2-sided upper and lower 95% confidence intervals (CIs) to assess the concordance between actual postoperative PFT, PPO-PFT estimated by ASC and CT volumetric analysis [13]. We used paired *t*-test to compare the difference of actual postoperative PFT and PPO-PFT estimated by ASC and CT volumetric analysis. All results were expressed as the mean ± standard deviation, or as proportions. In all analyses, a *P*-value of <0.05 was considered statistically significant.

## RESULTS

### Clinical characteristics of the study population

The clinical characteristics of the study population are summarized in Table 1. Ninety patients (51.4%) were male and 99 patients (56.6%) were never smoker. Most patient’s clinical stage is T1N0M0 (*N* = 155, 88.6%). One patient diagnosed with clinical stage T3 (satellite nodule in ipsilateral lobe) was included. The most of patients underwent 1 (*N* = 71, 40.6%) or 2 (*N* = 86, 49.1%) segments resected. The location of the tumour and the type of segmentectomy performed in the study population were described in Table 2. The upper division segmentectomy of the left upper lobe were the most frequently performed.

**Table 1:** Patient characteristics

Variables	*N* = 175
Sex (male) (*n*, %)	90 (51.4%)
Age (years)	62.9 ± 9.6
Smoking (*n*, %)	
Never smoker	99 (56.6%)
Ex-smoker	61 (34.8%)
Current smoker	15 (8.6%)
Clinical stage (TNM 8th edition) (*n*, %)	
T1 N0M0	155 (88.6%)
T2 N0M0	19 (10.8%)
T3 N0M0	1 (0.6%)
Number of resected segments (*n*, %)	
1	71 (40.6%)
2	86 (49.1%)
3	8 (4.6%)
4	10 (5.7%)
Reoperation (*n*, %)	15 (8.6%)
Segmentectomy (*n*, %)	
Simple	128 (73.1%)
Complex	47 (26.9%)
Baseline lung function	
FVC (l)	3.3 ± 0.8
FVC (% predicted)	99.0 ± 15.9
FEV_1_ (l)	2.4 ± 0.6
FEV_1_ (% predicted)	100.9 ± 22.1
FEV_1_/FVC, %	72.6 ± 11.1
D_LCO_ (mm/min/kPa)	16.9 ± 4.2
D_LCO_ (% predicted)	94.13 ± 19.06
Postoperative lung function	
FVC (l)	2.9 ± 1.8
FVC (% predicted)	89.3 ± 17.8
FEV_1_ (l)	2.1 ± 0.6
FEV_1_ (% predicted)	91.7 ± 21.0
FEV_1_/FVC (%)	73.2 ± 10.7
D_LCO_ (mm/min/kPa)	14.7 ± 3.9
D_LCO_ (% predicted)	83.6 ± 18.8

FVC: forced vital capacity; FEV_1_: forced expiratory volume in 1 s; D_LCO_: diffusing capacity for carbon monoxide.

**Table 2:** Location of resected segment

Variables	*N* = 175
RUL (*N* = 23; *n*, %)	
Apical	1 (0.6)
Posterior	8 (4.6)
Anterior	6 (3.4)
Apical + posterior	5 (2.9)
Apical + anterior	3 (1.7)
RLL (*N* = 47; *n*, %)	
Superior	22 (12.6)
Anterior basal	5 (2.9)
Posterior basal	3 (1.7)
Medial basal + anterior basal	2 (1.1)
Anterior basal + lateral basal	4 (2.3)
Superior + lateral basal + posterior basal	1 (0.6)
Common basal	10 (5.7)
LUL (*N* = 72; *n*, %)	
Apicoposterior	1 (0.6)
Anterior	2 (1.1)
Upper division	57 (32.6)
Lingula	12 (6.9)
LLL (*N* = 33; *n*, %)	
Superior	20 (11.4)
Superior + posterior basal	2 (1.1)
Anteromedial basal	2 (1.1)
Lateral basal + posterior basal	1 (0.6)
Anteromedial basal + lateral basal	1 (0.6)
Common basal	7 (4.0)

LLL: left lower lobe; LUL: left upper lobe; RLL: right lower lobe; RUL: right upper lobe.

### Concordance of predicted postoperative-pulmonary function test between anatomical segment counting and computed tomographic volumetric analysis

**Figure 1:**
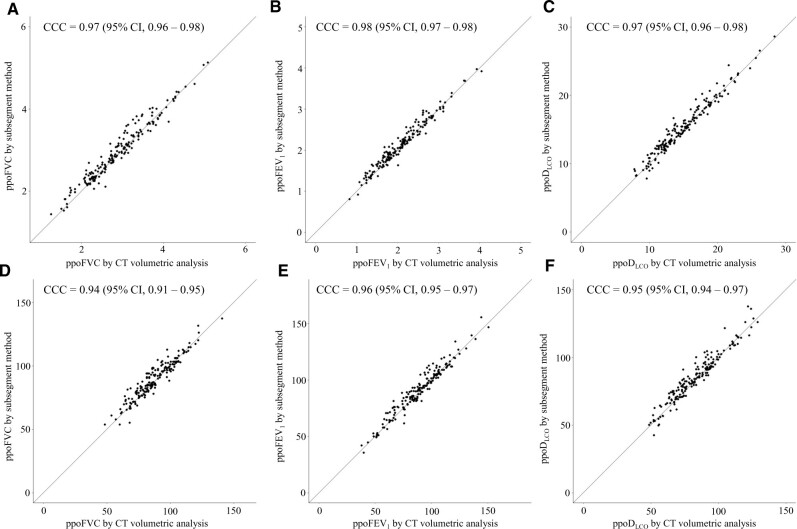
Concordance analysis of PPO-PFT between ASC and CT volumetric analysis. PPO-FVC (L) (**A**), PPO-FEV_1_ (L) (**B**), PPO-D_LCO_ (mm/min/kPa) (**C**), PPO-FVC (%) (**D**), PPO-FEV_1_ (%) (**E**) and PPO-D_LCO_ (%) (**F**) showed high concordance between CT volumetric analysis and ASC. ASC: anatomical segment counting; CCC: concordance correlation coefficient; CI: confidence interval; CT: computed tomography; D_LCO_: diffusing capacity for carbon monoxide; FEV_1_: forced expiratory volume in 1 s; FVC: forced vital capacity.

We analysed the concordance of PPO-PFT values estimated by ASC and CT volumetric analysis (Fig. 1). CT volumetric analysis showed high concordance with ASC for PPO-FVC (L) (CCC = 0.97, 95% CI 0.96–0.98), PPO-FVC (% predicted) (CCC = 0.94, 95% CI 0.91–0.95), PPO-FEV_1_ (L) (CCC = 0.98, 95% CI 0.97–0.98), PPO-FEV_1_ (% predicted) (CCC = 0.96, 95% CI 0.95–0.97), PPO-D_LCO_ (mm/min/kPa) (CCC = 0.97, 95% CI 0.96–0.98) and PPO-D_LCO_ (% predicted) (CCC = 0.95, 95% CI 0.94–0.97).

### Concordance of pulmonary function test between predicted value and actual postoperative value

**Figure 2:**
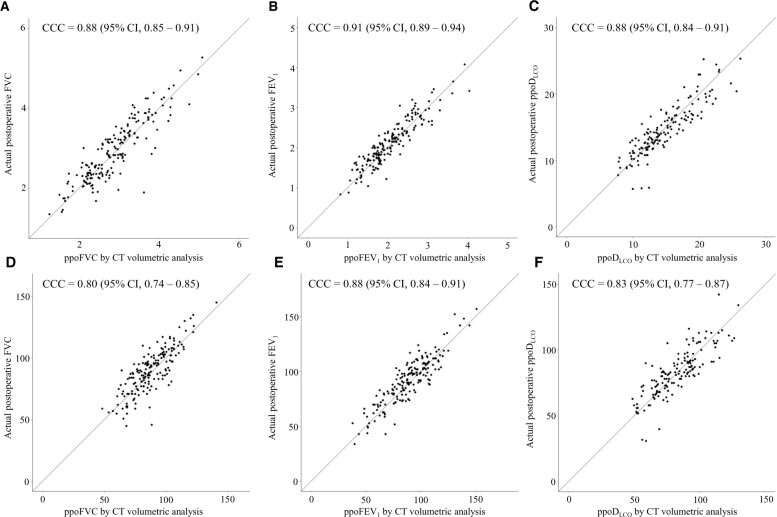
Concordance analysis between actual postoperative PFT values and PPO-PFT values calculated by ASC. PPO-FVC (L) (**A**), PPO-FEV_1_ (L) (**B**), PPO-D_LCO_ (mm/min/kPa) (**C**), PPO-FVC (%) (**D**), PPO-FEV_1_ (%) (**E**) and PPO-D_LCO_ (%) (**F**) showed high concordance. ASC: anatomical segment counting; CCC: concordance correlation coefficient; CI: confidence interval; CT: computed tomography; D_LCO_: diffusing capacity for carbon monoxide; FEV_1_: forced expiratory volume in 1 s; FVC: forced vital capacity.

PPO-PFT estimated by ASC showed high concordance with actual postoperative PFT for PPO-FVC (L) (CCC = 0.89, 95% CI 0.86–0.92), PPO-FVC (% predicted) (CCC = 0.82, 95% CI 0.77–0.86), PPO-FEV_1_ (L) (CCC = 0.93, 95% CI 0.90–0.95), PPO-FEV_1_ (% predicted) (CCC = 0.90, 95% CI 0.87–0.93), PPO-D_LCO_ (mm/min/kPa) (CCC = 0.88, 95% CI 0.84–0.91) and PPO-D_LCO_ (% predicted) (CCC = 0.83, 95% CI 0.78–0.88) (Fig. 2).

**Figure 3:**
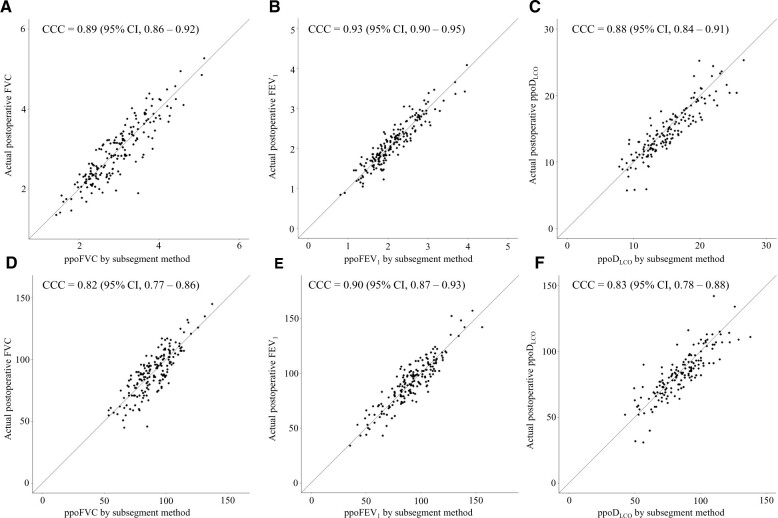
Concordance analysis between actual postoperative PFT values and PPO-PFT values calculated by CT volumetric analysis. PPO-FVC (L) (**A**), PPO-FEV_1_ (L) (**B**), PPO-D_LCO_ (mm/min/kPa) (**C**), PPO-FVC (%) (**D**), PPO-FEV_1_ (%) (**E**) and PPO-D_LCO_ (%) (**F**) showed high concordance. ASC: anatomical segment counting; CCC: concordance correlation coefficient; CI: confidence interval; CT: computed tomography; D_LCO_: diffusing capacity for carbon monoxide; FEV_1_: forced expiratory volume in 1 s; FVC: forced vital capacity.

PPO-PFT estimated by CT volumetric analysis also showed high concordance with actual postoperative PFT for PPO-FVC (L) (CCC = 0.88, 95% CI 0.85–0.91), PPO-FVC (% predicted) (CCC = 0.80, 95% CI 0.74–0.85), PPO-FEV_1_ (L) (CCC = 0.95, 95% CI 0.89–0.94), PPO-FEV_1_ (% predicted) (CCC = 0.88, 95% CI 0.84–0.91), PPO-D_LCO_ (mm/min/kPa) (CCC = 0.88, 95% CI 0.84–0.91) and PPO-D_LCO_ (% predicted) (CCC = 0.83, 95% CI 0.77–0.87) (Fig. 3).

Additionally, we compared the PPO-PFT estimated by ASC and CT volumetric analysis and actual postoperative PFT values by paired *t*-test. As a result, there was no statistically significant difference of all PFT values (Table 3).

**Table 3:** Comparison between actual postoperative and predicted postoperative PFT values estimated by 2 methods

Parameter	Actual value	ASC	CT volumetric analysis	*P*-value for actual versus ASC	*P*-value for actual versus CT	*P*-value for ASC versus CT
FVC (l)	2.9 ± 1.8	2.9 ± 1.8	2.9 ± 1.7	0.70	0.63	0.38
FVC (%)	89.3 ± 17.8	89.7 ± 15.0	87.5 ± 15.4	0.85	0.26	0.18
FEV_1_ (l)	2.1 ± 0.6	2.2 ± 0.6	2.1 ± 0.6	0.91	0.49	0.42
FEV_1_ (%)	91.7 ± 21.0	91.6 ± 20.5	89.2 ± 20.5	0.77	0.20	0.30
D_LCO_ (mm/min/kPa)	14.7 ± 3.9	15.3 ± 4.0	14.9 ± 4.1	0.15	0.55	0.39
D_LCO_ (%)	83.6 ± 18.8	85.0 ± 18.9	82.7 ± 18.6	0.41	0.80	0.25

ASC: anatomical segment counting; CT: computed tomography; DLCO: diffusing capacity for carbon monoxide; FEV1: forced expiratory volume in 1 s; FVC: forced vital capacity.

### Subgroup analysis

We subdivided the patients according to baseline PFT values, location of resected lobe and the number of segments to examine the predictability of both methods in certain group of patients.

We divided patients into 2 groups based on PFT values whether baseline FEV_1_ or D_LCO_ is <60%. The poor PFT group included 17 patients (9.7%) and normal PFT group included 158 patients (90.3%). We compared the PPO-FVC, PPO-FEV_1_, PPO-D_LCO_ measured by 2 methods and actual PFT values for each group. There was no statistically significant difference between PPO-PFT and actual PFT in both groups (Supplementary Material, Table S1). We also compared PPO-PFT and actual postoperative PFT values in subgroups, which were divided by the resected segment numbers. We categorized subgroups into 3 groups as follows: (i) one segment (*N* = 68, 38.9%), (ii) segments (*N* = 88, 50.3%) and (iii) 3 and 4 segments (*N* = 19, 10.9%). Conclusively, there was no statistically significant difference between PPO-PFT and actual PFT in all subgroups (Supplementary Material, Table S2). Lastly, we additionally compared PPO-PFT values according to the location of resected lobe and there was no statistically significant difference between PPO-PFT and actual PFT according to resected lobe (Supplementary Material, Table S3).

## DISCUSSION

This study demonstrated the predictability of PPO-PFT estimated by 2 methods in patients who underwent segmentectomy for early-stage lung cancer. The agreement between PPO-PFT estimated by ASC and CT volumetric analyses was quite high. Furthermore, the PPO-PFT values calculated using both methods did not differ significantly from the actual postoperative PFT values.

Before a major pulmonary resection, the ACCP recommends calculating PPO-PFT to evaluate the operative risk and determine the surgical extent. In lobectomy and pneumonectomy, a few alternative approaches for calculating PPO-PFT are advised. PPO-PFT should be assessed by a radionuclide perfusion scan in pneumonectomy and an ASC in lobectomy, according to the guidelines. They noted that the ASC method may be applied to segmentectomy because there would be no substantial difference in lung function decrease between segmentectomy and lobectomy [2]. Since lobectomy is currently the standard of care for surgical treatment of lung cancer, many methodologies for functional assessment of cardiopulmonary reserve have been presented. As the role of segmentectomy in the surgical treatment of lung cancer has been increased, precise prediction of pulmonary function in segmentectomy is also required.

Despite the fact that various studies have shown that PPO-PFT measurement using CT volumetric analysis is more accurate and better than ASC in major lung resection, our findings revealed that there was no statistically significant difference between the 2 approaches in segmentectomy [4,14]. Regardless of the resected lobe or the number of resected segments, both ASC and CT volumetric analyses performed well in predicting postoperative PFT. In the poor PFT group, our findings also revealed that there was no significant difference between the 2 methods. There might be 2 possible explanations for these outcomes. First, because the resected lung volume was small, it would be difficult to show a substantial difference in PPO-PFT. As previously stated, the majority of the resected segments are 1 (*N* = 71, 40.6%) or 2 (*N* = 86, 49.1%). Since the ratio of one lung segment is 1/19, ∼5.3%, the volume of the resected lung itself was small, it would be hard to make statistically difference. Second, CT volumetric analysis is a technique that uses anatomical references, such as the ASC. As a result, this method cannot reflect functional references such as lung perfusion scintigraphy or single-photon emission computed tomography/CT [14].

### Limitations

There are some limitations of this study that should be addressed. First, it should be noted that this is a single-centre observational study. These results should be confirmed by a multicentre or prospective study because their generalizability to different populations may be limited. Second, the impact of the severity of any underlying pulmonary disease was not assessed. It was merely a volume comparison, with the drawback that it did not assess the functional component. Third, due to the retrospective nature of the study, the CT acquisition protocol was not uniform across all subjects and there is the possibility of uncontrolled bias. Fourth, the study population underwent surgery before our centre adopted near-infrared fluorescence imaging for identifying intersegmental planes. We used a ventilation method that may not be as accurate as the near-infrared method, therefore the PPO- and actual PFT values may be affected.

## CONCLUSION

Our results demonstrated that both ASC and CT volumetric analysis performed well in evaluating PPO-FEV1 and PPO-DLCO in segmentectomy for early-stage lung cancer. This finding indicated that ASC, which was previously thought to be the standard approach, could accurately predict postoperative PFT.

## SUPPLEMENTARY MATERIAL

Supplementary material is available at *ICVTS* online.

## Supplementary Material

ivac195_Supplementary_Data

## Data Availability

The data underlying this article will be shared on reasonable request to the corresponding author.

## ABBREVIATIONS

ACCP: American College of Chest Physicians
ASC: Anatomical segment counting
CCC: Concordance correlation coefficient
CI: Confidence interval
CT: Computed tomography
D_LCO_: Diffusing capacity for carbon monoxide
FEV_1_: Forced expiratory volume in 1 s
FVC: Forced vital capacity
PFT: Pulmonary function test
PPO: Predicted postoperative
