# Supplementary material for: Computed tomography volumetric analysis for predicting postoperative lung function for segmentectomy
Source: Interact Cardiovasc Thorac Surg. 2022 Jul 25;35(2):ivac195. doi: 10.1093/icvts/ivac195 (PMC9338704; doi:10.1093/icvts/ivac195)
Supplement: ivac195_Supplementary_Data [file ivac195_supplementary_data.docx]

**Supplementary Table S1. Subgroup analysis about normal and poor PFT groups**

| Groups | Parameter | Actual value | ASC | CT volumetric  analysis | P-value for  actual vs. ASC | P-value for  actual vs. CT | P-value for  ASC vs. CT |
| --- | --- | --- | --- | --- | --- | --- | --- |
| Normal PFT (N=158) | FVC, % predicted | 90.7 ± 17.1 | 90.7 ± 14.0 | 88.4 ± 14.8 | 0.97 | 0.19 | 0.14 |
|  | FEV_1_, % predicted | 94.7 ± 19.5 | 94.3 ± 18.0 | 91.8 ± 18.4 | 0.87 | 0.18 | 0.22 |
|  | D_LCO_, % predicted | 85.5 ± 18.1 | 87.4 ± 16.7 | 85.0 ± 16.8 | 0.33 | 0.83 | 0.20 |
| Poor PFT (N=17) | FVC, % predicted | 78.0 ± 21.1 | 79.2 ± 20.0 | 79.1 ± 18.5 | 0.87 | 0.87 | 0.99 |
|  | FEV_1_, % predicted | 67.1 ± 23.1 | 63.7 ± 22.5 | 64.0 ± 22.4 | 0.67 | 0.69 | 0.98 |
|  | D_LCO_, % predicted | 66.8 ± 17.3 | 64.8 ± 14.3 | 64.9 ± 13.8 | 0.73 | 0.74 | 0.99 |

ASC, anatomical segment counting; CT, computed tomography; PFT, pulmonary function test; FVC, forced vital capacity; FEV_1_, forced expiratory volume in 1 second; D_LCO_, diffusing capacity for carbon monoxide.

Supplementary Table S2. Subgroup analysis based on number of resected segments

| Segment numbers | Parameter | Actual value | ASC | CT volumetric  analysis | P-value for  actual vs. ASC | P-value for  actual vs. CT | P-value for  ASC vs. CT |
| --- | --- | --- | --- | --- | --- | --- | --- |
| 1 (N=68) | FVC, % predicted | 90.3 ± 19.2 | 92.4 ± 15.9 | 91.6 ± 16.5 | 0.47 | 0.67 | 0.76 |
|  | FEV_1_, % predicted | 92.5 ± 21.7 | 94.0 ± 20.1 | 93.2 ± 20.6 | 0.67 | 0.85 | 0.80 |
|  | D_LCO_, % predicted | 85.0 ± 15.0 | 57.6 ± 15.9 | 86.7 ± 16.2 | 0.35 | 0.54 | 0.75 |
| 2 (N=88) | FVC, % predicted | 90.3 ± 16.6 | 90.0 ± 13.7 | 85.9 ± 14.4 | 0.91 | 0.06 | 0.05 |
|  | FEV_1_, % predicted | 93.2 ± 20.7 | 92.0 ± 20.7 | 87.8 ± 20.6 | 0.70 | 0.08 | 0.18 |
|  | D_LCO_, % predicted | 84.4 ± 20.3 | 85.9 ± 17.9 | 81.9 ± 17.9 | 0.64 | 0.40 | 0.15 |
| 3, 4 (N=19) | FVC, % predicted  FEV_1_, % predicted  D_LCO_, % predicted | 83.1 ± 17.9 | 78.2 ± 12.3 | 80.2 ± 11.8 | 0.33 | 0.56 | 0.60 |
|  |  | 84.8 ± 23.3 | 79.0 ± 17.7 | 80.9 ± 17.0 | 0.39 | 0.56 | 0.73 |
|  |  | 75.0 ± 22.2 | 73.9 ± 19.5 | 75.4 ± 17.5 | 0.88 | 0.96 | 0.81 |

ASC, anatomical segment counting; CT, computed tomography; FVC, forced vital capacity; FEV_1_, forced expiratory volume in 1 second; D_LCO_, diffusing capacity for carbon monoxide.

Supplementary Table S3. Subgroup analysis based on location of segment

| Location of segment | Parameter | Actual value | ASC | CT volumetric  analysis | P-value for  actual vs. ASC | P-value for  actual vs. CT | P-value for  ASC vs. CT |
| --- | --- | --- | --- | --- | --- | --- | --- |
| RUL (N=23) | FVC, % predicted | 91.6 ± 18.6 | 90.7 ± 14.5 | 88.8 ± 15.0 | 0.86 | 0.57 | 0.65 |
|  | FEV_1_, % predicted | 93.6 ± 24.5 | 91.5 ± 21.0 | 89.6 ± 21.7 | 0.76 | 0.56 | 0.77 |
|  | D_LCO_, % predicted | 76.7 ± 11.5 | 81.9 ± 13.5 | 80.1 ± 13.3 | 0.20 | 0.40 | 0.64 |
| RLL (N=47) | FVC, % predicted | 89.8 ± 16.4 | 88.9 ± 15.1 | 89.5 ± 14.3 | 0.78 | 0.92 | 0.84 |
|  | FEV_1_, % predicted | 92.2 ± 18.7 | 90.9 ± 19.6 | 91.4 ± 8.5 | 0.74 | 0.83 | 0.91 |
|  | D_LCO_, % predicted | 80.7 ± 18.3 | 82.0 ± 16.7 | 82.3 ± 15.7 | 0.75 | 0.67 | 0.91 |
| LUL (N=72) | FVC, % predicted  FEV_1_, % predicted  D_LCO_, % predicted | 89.4 ± 17.2 | 90.8 ± 14.3 | 85.5 ± 15.2 | 0.59 | 0.16 | 0.33 |
|  |  | 92.5 ± 20.9 | 92.8 ± 20.6 | 87.4 ± 20.8 | 0.93 | 0.15 | 0.12 |
|  |  | 84.2 ± 21.5 | 86.3 ± 19.0 | 81.1 ± 18.8 | 0.55 | 0.39 | 0.11 |
| LLL (N=33) | FVC, % predicted | 87.8 ± 21.0 | 87.5 ± 17.1 | 88.0 ± 17.3 | 0.95 | 0.97 | 0.91 |
|  | FEV_1_, % predicted | 89.6 ± 24.6 | 88.9 ± 22.0 | 89.4 ± 22.2 | 0.90 | 0.96 | 0.93 |
|  | D_LCO_, % predicted | 89.9 ± 15.3 | 90.0 ± 18.3 | 90.5 ± 18.4 | 0.99 | 0.89 | 0.91 |

ASC, anatomical segment counting; CT, computed tomography; RUL, right upper lobe; RLL, right lower lobe; LUL, left upper lobe; LLL, left lower lobe; FVC, forced vital capacity; FEV_1_, forced expiratory volume in 1 second; D_LCO_, diffusing capacity for carbon monoxide.
